# The effect of the surgical helmet system on intraoperative contamination in arthroplasty surgery: a fluorescent quantitative simulation

**DOI:** 10.1302/2633-1462.411.BJO-2023-0078.R1

**Published:** 2023-11-13

**Authors:** Hongtai Chen, Vincent W. K. Chan, Chun H. Yan, Henry Fu, Ping-Keung Chan, KwongYuen Chiu

**Affiliations:** 1 Division of Joint Replacement Surgery, Department of Orthopaedics and Traumatology, The University of Hong Kong, Queen Mary Hospital, Hong Kong, China

**Keywords:** Arthroplasty, Periprosthetic joint infection, Surgical helmet system, Body exhaust system, Quantitative simulation, Glove-gown interface, arthroplasty surgeries, periprosthetic joint infection (PJI), standard deviations, clinical study, infections, ANOVA, bone resection, correlation coefficient, Statistical analysis, bacteria

## Abstract

**Aims:**

The surgical helmet system (SHS) was developed to reduce the risk of periprosthetic joint infection (PJI), but the evidence is contradictory, with some studies suggesting an increased risk of PJI due to potential leakage through the glove-gown interface (GGI) caused by its positive pressure. We assumed that SHS and glove exchange had an impact on the leakage via GGI.

**Methods:**

There were 404 arthroplasty simulations with fluorescent gel, in which SHS was used (H+) or not (H-), and GGI was sealed (S+) or not (S-), divided into four groups: H+S+, H+S-, H-S+, and H-S-, varying by exposure duration (15 to 60 minutes) and frequency of glove exchanges (0 to 6 times). The intensity of fluorescent leakage through GGI was quantified automatically with an image analysis software. The effect of the above factors on fluorescent leakage via GGI were compared and analyzed.

**Results:**

The leakage intensity increased with exposure duration and frequency of glove exchanges in all groups. When SHS was used and GGI was not sealed (H+S-), the leakage intensity via GGI had the fastest increase, consistently higher than other groups (H+S+, H-S+ and H-S-) after 30 minutes (p < 0.05) and when there were more than four instances of glove exchange (p < 0.05). Additionally, the leakage was strongly correlated with the duration of exposure (r_s_ = 0.8379; p < 0.050) and the frequency of glove exchange (r_s_ = 0.8198; p < 0.050) in H+S-. The correlations with duration and frequency turned weak when SHS was not used (H-) or GGI was sealed off (S+).

**Conclusion:**

Due to personal protection, SHS is recommended in arthroplasties. Meanwhile, it is strongly recommended to seal the GGI of the inner gloves and exchange the outer gloves hourly to reduce the risk of contamination from SHS.

Cite this article: *Bone Jt Open* 2023;4(11):859–864.

## Introduction

In the early days of arthroplasty surgery, the infection rate was as high as 9.5%.^1^ However, with advances in surgical hygiene and infection control measures, the rate has decreased to approximately 1% to 2%.^2^ It has been found that up to 98% of infections are caused by airborne microbial contaminations,^3^ and the primary source of this contamination is from personnel in the surgical theatre, particularly surgeons.^4^ Therefore, it is important to prevent microbial contamination by surgeons.

In recent years, a positive pressure surgical helmet system (SHS) has been developed as a simpler alternative to the negative pressure body exhaust system (BES) for preventing surgeon-derived contamination in arthroplasty surgery.^5^ Although many surgeons view SHS as the successor of BES, their mechanisms of action are diametrically opposed. With SHS, a ventilated helmet pumps air into the gown and hood, creating a positive pressure that can lead particle leakage through various gaps with low resistance in the gown.^6^ This positive pressure raises concerns about potential contaminated particles from the collar-hood and glove-gown interfaces (GGI).^7,8^ Moreover, some studies have suggested that SHS only protects surgeons and other scrubbed personnel from potential fluid and bloodborne transmissions,^9^ but does not reduce the incidence of surgical field contaminations.^10-14^ As a result, the effectiveness of SHS in reducing the risk of surgeon-derived contamination and PJI has been questioned.

In this study, a fluorescent quantitative simulation was designed to investigate the risk of contaminated particle leakage via the GGI under various settings, including the use of SHS, sealing of GGI, duration of exposure, and frequency of glove exchange. Our hypothesis was that the positive pressure caused by SHS would increase the risk of particle leakage, especially with more frequent glove exchanges, while the sealing of the GGI would reduce it.

## Methods

**Fig. 1:**
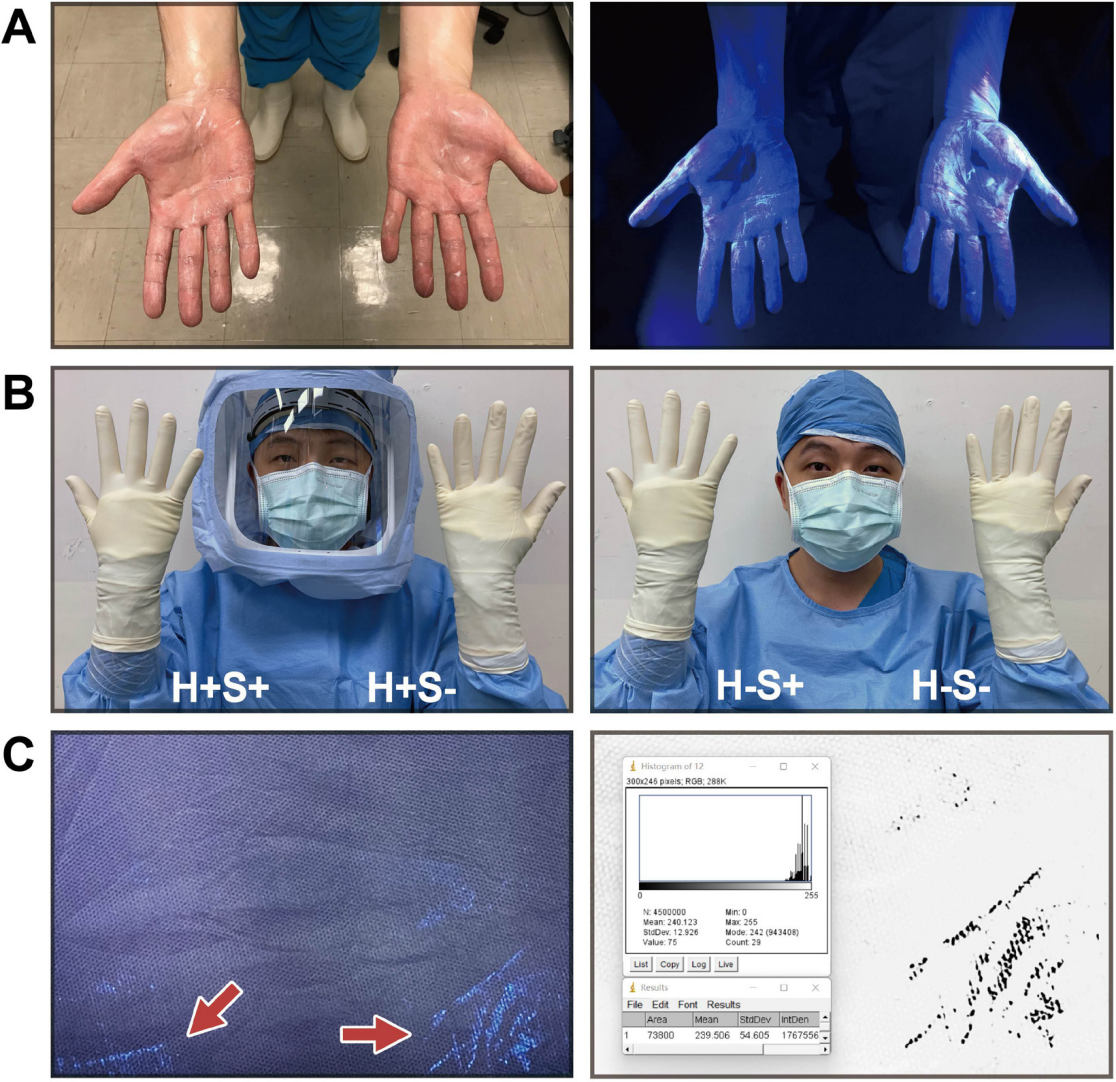
Method for fluorescent quantitative simulation. a) Hand scrubbing with fluorescent gel and fluorescent signal under ultraviolet (UV) light. b) Four specific groups for simulations (H+S+, H+S-, H-S+, and H-S-). c) Fluorescent leakage under UV light (red arrow) and fluorescent leakage intensity quantification (blue arrow).

This is a simulation study, and institutional review board approval has been waived. The fluorescent quantitative simulation was performed with ultraviolet fluorescent gel (Glo Germ, USA), which contains fluorescent particlescomparable to the size of a bacterium (5 μm in diameter) to simulate contaminated particles on the skin (Figure 1a).We aim to investigate the degree of fluorescent leakage quantitatively under different circumstances, such as the use of SHS, the sealing of GGI, the duration of exposure, and the frequency of glove exchange.

After putting on a surgical gown (3M, USA) with or without SHS (Flyte; Stryker, USA), the surgeon scrubbed his hands with five drops (about 0.25 ml) of fluorescent gel simulating residual bacteria on the skin. After air-drying both hands, the surgeon put on a pair of powder-free gloves (Gammex, Ansell, Australia). The right GGI was sealed with surgical tape (3M), while the left was not sealed. Both hands are double-gloved, following our routine practice. An assistant then used a 365 nm ultraviolet (UV) lamp to ensure there was no fluorescent leakage before the start of the simulations.

The simulations were divided into four groups (Figure 1b), including SHS with tape sealing GGI (H+S+), SHS without sealing GGI (H+S-), no SHS with tape sealing GGI (H-S+), and no SHS or tape (H-S-). The surgeon then used an empty saw with each hand for five minutes to simulate the bone resection during arthroplasty. The surgeon changed the outer gloves from zero to six times during the simulation. After excluding invalid simulations, there were a total of 404 simulations analyzed in the study, including 107, 100, 99, and 98 simulations in H+S+, H+S-, H-S+, and H-S-, respectively. All simulations were performed on the same test subject to standardize the scrubbing, gowning, gloving, and glove exchange techniques.

At various time intervals (15, 30, 45, and 60 minutes), all sleeves were removed and flattened for photographs under UV light in the dark by Canon 5D2 (Canon, Japan) with the same angle, distance, and camera settings. The fluorescent leakage intensity of the area above GGI was quantified by the image analysis software (ImageJ, National Institutes of Health, USA) and expressed as an integrated density value (IDV), representing the sum of the intensity values of the target pixels in the image (Figure 1c).

### Statistical analysis

Statistical analysis was performed using SPSS Statistics 26 (IBM, USA). The IDVs obtained from different simulation scenarios and timepoints were compared using a two-way analysis of variance (ANOVA) test, followed by a Tukey post-hoc test to identify significant differences. To assess the correlation between IDV and independent variables, such as duration of exposure and frequency of glove exchanges, Spearman’s rank correlation coefficient (r_s_) was calculated for each group. A r_s_-value less than 0.4, between 0.4 and 0.6, between 0.6 and 0.8, and greater than 0.8 were considered weak, moderate, strong, and very strong correlations, respectively. A p-value less than 0.05 was considered statistically significant.

## Results

**Fig. 2:**
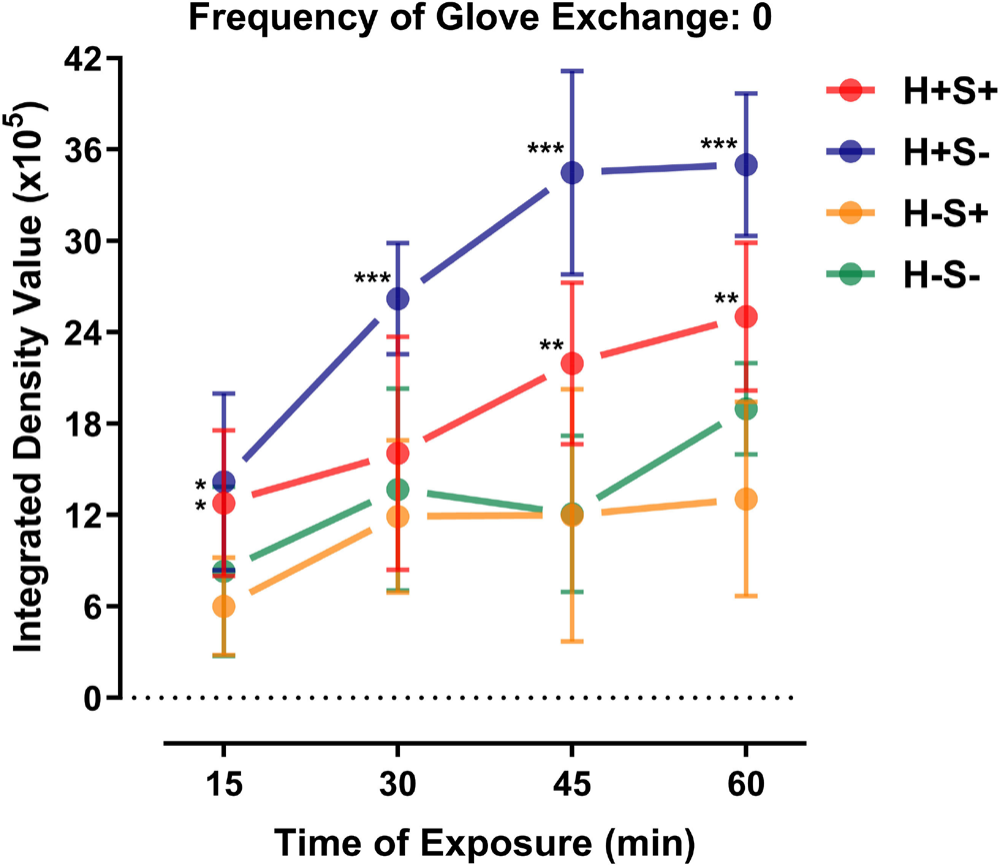
Integrated density values of different simulation scenarios with time (minutes). *Significant difference from H-S+ (p < 0.05). **Significant difference from H-S- and H-S+ (p < 0.05). ***Significant difference from H+S+, H-S-, and H-S+ (p < 0.05).

When the frequency of glove exchange was fixed at 0, our results showed that the different simulation scenarios and duration of exposure had an effect on the IDVs of fluorescent leakage via GGI (p < 0.001, two-way ANOVA) with no interactions using two-way ANOVA (Figure 2 and Table I). Post-hoc LSD test revealed that the IDV of the SHS without sealing GGI (H+S-) was higher than the other three simulation scenarios from 30 minutes to 60 minutes (p < 0.050). The mean IDV at 45 minutes of H+S-, H+S+, H-S-, and H-S+ was 34.49 (standard deviation (SD) 5.79; 95% confidence intervals (CIs) 26.13 to 40.99), 21.96 (SD 4.84; 95% CI 13.49 to 28.39), 12.08 (SD 4.60; CI 7.17 to 19.08), and 12.00 (SD 7.40; CI 4.28 to 25.71) respectively (Table I). The IDV of H+S- was higher than the two groups without SHS (H-S+ and H-S-) at 15 minutes (p < 0.050, Figure 2). The IDV of the SHS with tape sealing GGI (H+S+) was greater than H-S+ at 15 minutes, and greater than both H-S- and H-S+ at 45 and 60 minutes (p < 0.050) (Figure 2). There was no difference in IDV between the two simulation scenarios without SHS (H-S+ and H-S-). The correlations between duration of exposure and IDV were very strong in H+S- (r_s_ = 0.8379; p = 0.001), strong in H+S+ (r_s_ = 0.6584; p = 0.001), and moderate in H-S- (r_s_ = 0.5770; p = 0.006), while there was no correlation in H-S+ (p = 0.068) (Table II).

**Table I.** Integrated density value of the different simulation scenarios at different timepoints without glove exchange. All values are presented as means with standard deviations and 95% confidence intervals.

Time	Simulation scenarios			
	H+S+	H+S-	H-S+	H-S-
15 mins	12.79 (4.42; 4.69 to 16.67)	14.18 (5.3; 7.15 to 21.95)	6.01 (2.78; 2.87 to 10.12)	8.31 (5.18; 1.03 to 16.62)
30 mins	16.07 (6.99; 7.76 to 30.39)	26.21 (3.17; 21.9 to 30.46)	11.91 (4.34; 7.25 to 18.54)	13.7 (5.74; 7.12 to 21.3)
45 mins	21.96 (4.84; 13.49 to 28.39)	34.49 (5.79; 26.13 to 40.99)	12 (7.4; 4.28 to 25.71)	12.08 (4.6; 7.17 to 19.08)
60 mins	25.04 (4.19; 19.57 to 31.16)	35.02 (4.28; 28.85 to 40.46)	13.05 (5.53; 4.51 to 19.97)	18.98 (2.61; 15.28 to 22.64)

H-, surgical helmet system was not used; H+, surgical helmet system was used; S-, glove-gown interface was not sealed; S+, glove-gown interface was sealed.

**Table II.** Summary of the correlation between the different simulation scenarios and integrated density values.

Scenario	Sealed GGI		Unsealed GGI	
	Duration of exposure	Frequency of glove exchanges	Duration of exposure	Frequency of glove exchanges
SHS	Strong correlation (r_s_ = 0.6584)	Not significant	Very strong correlation (r_s_ = 0.8379)	Very strong correlation (r_s_ = 0.8198)

No SHS	Not significant	Moderate correlation (r_s_ = 0.4850)	Moderate correlation (r_s_ = 0.5770)	Moderate correlation (r_s_ = 0.5816)

GGI, glove-gown interface; SHS, surgical helmet system.

**Fig. 3:**
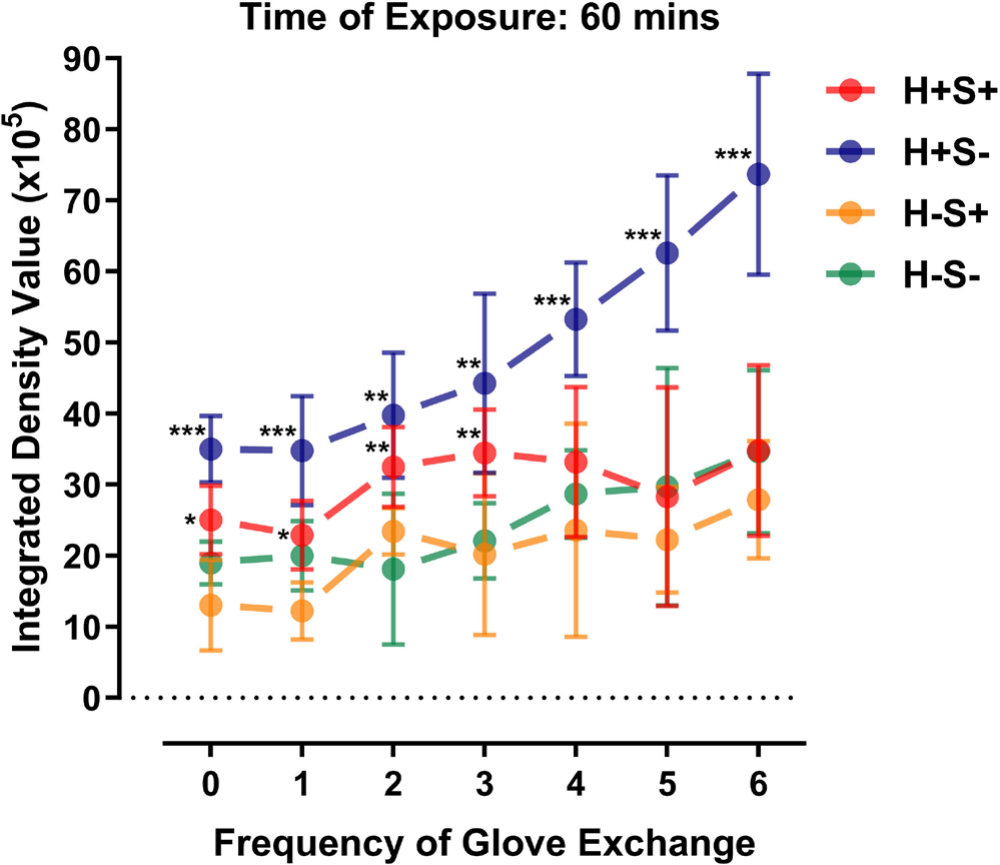
Integrated density values of different simulation scenarios at various frequency of glove exchanges. *Significant difference from H-S+ (p < 0.05). **Significant difference from H-S- and H-S+ (p < 0.05). ***Significant difference from H+S+, H-S+, and H-S- (p < 0.05).

When the duration of exposure was fixed at 60 minutes, our results showed that the different simulation scenarios and frequency of glove exchange had an effect on the integrated density values (IDVs) of fluorescent leakage via GGI (p < 0.001) without any interactions (Figure 3 and Table III). Post-hoc LSD test revealed that the SHS without sealing GGI (H+S-) had higher IDVs than the other three simulation scenarios when the frequency of glove exchanges were zero, one, four, five, and six (p < 0.050), and was higher than the two simulation scenarios without SHS at two and three glove exchanges (p < 0.050) (Figure 3). At four glove exchanges, the mean IDV of H+S-, H+S+, H-S-, and H-S+ was 53.30 (SD 6.91; CI 45.91 to 64.28), 33.16 (SD 8.65; CI 21.52 to 42.24), 28.71 (SD 0.12; CI 28.58 to 28.83), and 23.62 (SD 16.36; CI 0.83 to 38.47), respectively (Table III). The SHS with tape sealing GGI (H+S+) had higher IDVs than H-S+ from zero to three glove exchanges (p < 0.050), and was greater than H-S- at two and three glove exchanges (p < 0.050). There was no difference in IDV between H-S+ and H-S- (p > 0.050). The correlations between frequency of glove exchange and IDV was very strong in H+S- (r_s_ = 0.8198, p < 0.05) and turned moderate in H-S+ and H-S- (r_s_ = 0.4850 and 0.5816, respectively, p < 0.05), while there was no correlation in H+S+ (p > 0.05) (Table II).

**Table III.** Integrated density value of the different simulation scenarios with different glove exchange frequencies at 60 minutes. All values are presented as means with standard deviations and 95% confidence intervals.

Frequency	Simulation scenarios			
	H+S+	H+S-	H-S+	H-S-
0	25.04 (4.19; 19.57 to 31.16)	35.02 (4.28; 28.85 to 40.46)	13.05 (5.53; 4.51 to 19.97)	18.98 (2.61; 15.28 to 22.64)
1	22.89 (4.4; 18.55 to 29.75)	34.8 (SD 6.66; 25.52 to 42.81)	12.26 (3.52; 9.75 to 18.33)	20.03 (4.24; 14.3 to 25.92)
2	32.48 (4.87; 24.75 to 38.21)	39.79 (3.3; 35.27 to 44.24)	23.42 (2.84; 18.64 to 25.64)	18.15 (9.49; 5.22 to 32.55)
3	34.45 (1.83; 32.26 to 36.99)	44.27 (10.9; 26.66 to 55.66)	20.21 (10.36; 2.44 to 31.71)	22.12 (4.36; 15.97 to 25.63)
4	33.16 (8.65; 21.52 to 42.24)	53.3 (6.91; 45.91 to 64.28)	23.62 (16.36; 0.83 to 38.47)	28.71 (0.12; 28.58 to 28.83)
5	28.31 (18.86; 8.9 to 53.08)	62.57 (9.51; 49.87 to 76.27)	22.28 (6.11; 15.43 to 30.27)	29.68 (14.96; 1.4 to 41.49)
6	34.79 (9.8; 22.92 to 46.92)	73.7 (10.02; 63.69 to 83.72)	27.87 (6.74; 18.72 to 34.76)	34.63 (9.37; 26.2 to 47.71)

H-, surgical helmet system was not used; H+, surgical helmet system was used; S-, glove-gown interface was not sealed; S+, glove-gown interface was sealed.

## Discussion

In this study, we used fluorescent quantitative simulation to investigate the effects of SHS and the sealing of GGI on particle leakage during arthroplasty surgery. Our findings showed that, regardless of the duration of exposure or frequency of glove changes, particle leakage via GGI was the greatest when SHS was used without GGI sealing (H+S-), and was substantially reduced after proper sealing of the GGI. These results support our hypothesis that the positive pressure from SHS increases the risk of contaminant leakage via GGI. However, we acknowledge that surgical splash is an inherent risk in arthroplasty surgeries, exposing surgeons and other scrub personnel to potential fluid and bloodborne transmissions. Despite the risk of particle leakage via the GGI, the use of SHS is strongly recommended in arthroplasty to protect scrub personnel from infectious agents.^15^ Therefore, we propose that GGI sealing with SHS can be an effective strategy to eliminate the impact of positive pressure and reduce the risk of contamination.

The IDV of fluorescent leakage via GGI increased significantly with the duration of exposure and the frequency of glove exchange. The strongest correlation was observed when SHS was used and GGI was not sealed (H+S-), indicating that longer surgery duration or more frequent glove exchanges could increase the risk of GGI-derived contamination. These results were contrary to the generally accepted consensus that glove exchanges can reduce the risk of PJI in arthroplasties.^16^ We speculate that the motion in the GGI during each glove exchange could explain our findings, as it increases the chance of particle leakage, especially in a positive pressure system like SHS. Furthermore, our findings suggest that sealing off the GGI with tape could reduce the risk of particle leakage during glove exchanges, as the correlation between IDV and the number of glove exchanges turned from very strong to insignificant after GGI sealing. Additionally, sealing off the GGI while using SHS reduced the correlation between IDV and exposure duration from very strong (r_s_ = 0.839) to strong (r_s_ = 0.658). A summary of our findings is presented in Table II.

Based on current literature, only one clinical study has examined the effect of sealing the GGI in the context of SHS and arthroplasty. Shirley et al^17^ randomized 75 patients into three groups: standard surgical gown, SHS without sealing the GGI, and SHS with sealed GGI. They found no significant difference in positive wound culture; however, the overall contamination rate was lower than expected, and the possibility of a type II error was raised. Notably, the study did not report whether the surgeon used a single or double gloving technique, or the frequencies of glove exchanges. In contrast, our simulation study showed that sealing the GGI reduces fluorescent leakage, particularly with increasing numbers of glove exchanges. Thus, these factors may have affected the results of the clinical study by Shirley et al. A larger-scale clinical study is warranted to investigate the potential benefit of GGI sealing in reducing contamination and PJI when using SHS during arthroplasties.

It is important to note that there are limitations to this fluorescent quantitative simulation study. In each simulation, the sleeves had to be cut off and flattened in order to quantify the IVD at the specific timepoints. As a result, the IDVs between different timepoints were treated as independent samples rather than a continuous simulation. While this approach enabled us to indirectly reflect the change of IDV with time, it cannot directly quantify it. Moreover, the IDV over the surgical field was not examined in this simulation study. Measuring potential contamination in the surgical field, such as around surgical drapes, would be interesting and beneficial for future research.

Despite the limitations, our study provides important insights into the potential risks of particle leakage via GGI during arthroplasty surgery, and highlights the importance of taking additional precautions to prevent contaminations. Further studies are needed to validate our findings, and explore other factors and strategies in minimizing the risk of contamination in the context of SHS and arthroplasty.

To conclude, the use of SHS in arthroplasty is important for personal protection, despite the risk of particle leakage via GGI. The risk can be minimized by reducing exposure duration and sealing the GGI of the inner gloves.

**Take home message**

- The use of a surgical helmet system in arthroplasty is important for personal protection, despite the risk of particle leakage via the glove-gown interface (GGI).

- This risk can be minimized by reducing exposure duration, and sealing the GGI of the inner gloves.

## References

[1] Charnley J A clean-air operating enclosure Br J Surg 1964 51 202 205 10.1002/bjs.1800510309 14129434

[2] Kurtz SM Lau E Watson H Schmier JK Parvizi J Economic burden of periprosthetic joint infection in the United States J Arthroplasty 2012 27 8 Suppl 61 65 10.1016/j.arth.2012.02.022 22554729

[3] Whyte W Hodgson R Tinkler J The importance of airborne bacterial contamination of wounds J Hosp Infect 1982 3 2 123 135 10.1016/0195-6701(82)90004-4 6181129

[4] Owers KL James E Bannister GC Source of bacterial shedding in laminar flow theatres J Hosp Infect 2004 58 3 230 232 10.1016/j.jhin.2004.06.028 15501339

[5] No authors listed Surgical helmet systems Health Devices 1996 25 4 116 145 10.1177/183335839602500417 8722101

[6] Ling F Halabi S Jones C Comparison of air exhausts for surgical body suits (space suits) and the potential for periprosthetic joint infection J Hosp Infect 2018 99 3 279 283 10.1016/j.jhin.2018.03.012 29559232

[7] McGovern PD Albrecht M Khan SK Muller SD Reed MR The influence of surgical hoods and togas on airborne particle concentration at the surgical site: an experimental study J Orthop Sci 2013 18 6 1027 1030 10.1007/s00776-013-0445-7 23943223

[8] Fraser JF Young SW Valentine KA Probst NE Spangehl MJ The gown-glove interface is a source of contamination: A comparative study Clin Orthop Relat Res 2015 473 7 2291 2297 10.1007/s11999-014-4094-8 25488405 PMC4457760

[9] No authors listed Personal Protection System: Information for Healthcare Professionals Stryker 2022 https://www.stryker.com/us/en/orthopaedic-instruments/products/flyte-personal-protection-system.html date last accessed 9 October 2023

[10] Pasquarella C Pitzurra O Herren T Poletti L Savino A Lack of influence of body exhaust gowns on aerobic bacterial surface counts in a mixed-ventilation operating theatre. A study of 62 hip arthroplasties J Hosp Infect 2003 54 1 2 9 10.1016/s0195-6701(03)00077-x 12767840

[11] Der Tavitian J Ong SM Taub NA Taylor GJS Body-exhaust suit versus occlusive clothing. A randomised, prospective trial using air and wound bacterial counts J Bone Joint Surg Br 2003 85-B 4 490 494 10.1302/0301-620x.85b4.13363 12793550

[12] Hooper GJ Rothwell AG Frampton C Wyatt MC Does the use of laminar flow and space suits reduce early deep infection after total hip and knee replacement?: the ten-year results of the New Zealand Joint Registry J Bone Joint Surg Br 2011 93-B 1 85 90 10.1302/0301-620X.93B1.24862 21196549

[13] Ghazavi MT Role of body exhaust system in reducing peri-prosthetic joint infection, literature review J Res Orthop 2016 3 e8568 10.17795/soj-8568

[14] Young SW Zhu M Shirley OC Wu Q Spangehl MJ Do “surgical helmet systems” or “body exhaust suits” affect contamination and deep infection rates in arthroplasty? A systematic review J Arthroplasty 2016 31 1 225 233 10.1016/j.arth.2015.07.043 26321627

[15] Makovicka JL Bingham JS Patel KA Young SW Beauchamp CP Spangehl MJ Surgeon personal protection: An underappreciated benefit of positive-pressure exhaust suits Clin Orthop Relat Res 2018 476 6 1341 1348 10.1007/s11999.0000000000000253 29481350 PMC6263565

[16] Kim K Zhu M Munro JT Young SW Glove change to reduce the risk of surgical site infection or prosthetic joint infection in arthroplasty surgeries: a systematic review ANZ J Surg 2019 89 9 1009 1015 10.1111/ans.14936 30497094

[17] Shirley OC Bayan A Zhu M Dalton JP Wiles S Young SW Do surgical helmet systems affect intraoperative wound contamination? A randomised controlled trial Arch Orthop Trauma Surg 2017 137 11 1565 1569 10.1007/s00402-017-2795-7 28918534

